# Risk Factors for Pulmonary Embolism in Patients with Paralysis and Deep Venous Thrombosis

**DOI:** 10.3390/jcm10225412

**Published:** 2021-11-19

**Authors:** Karsten Keller, Jens Wöllner, Volker H. Schmitt, Mir A. Ostad, Ingo Sagoschen, Thomas Münzel, Christine Espinola-Klein, Lukas Hobohm

**Affiliations:** 1 Department of Cardiology, Cardiology I, University Medical Center Mainz (Johannes Gutenberg-University Mainz), 55131 Mainz, Germany; Volker.Schmitt@unimedizin-mainz.de (V.H.S.); Ostad@uni-mainz.de (M.A.O.); Ingo.Sagoschen@unimedizin-mainz.de (I.S.); tmuenzel@uni-mainz.de (T.M.); espinola@uni-mainz.de (C.E.-K.); lukas.hobohm@unimedizin-mainz.de (L.H.); 2 Center for Thrombosis and Hemostasis (CTH), University Medical Center Mainz (Johannes Gutenberg-University Mainz), 55131 Mainz, Germany; 3 Medical Clinic VII, Department of Sports Medicine, University Hospital Heidelberg, 69120 Heidelberg, Germany; 4 Swiss Paraplegic Center Nottwil, Department of Neuro-Urology, 6207 Nottwil, Switzerland; jens.woellner@paraplegie.ch; 5 Department of Urology and Pediatric Urology, University Medical Center Mainz (Johannes Gutenberg-University Mainz), 55131 Mainz, Germany; 6 German Center for Cardiovascular Research (DZHK), Partner Site Rhine Main, 55131 Mainz, Germany

**Keywords:** paralysis, stroke, mortality, spinal cord injury, pulmonary embolism

## Abstract

Background. Venous thromboembolism is a frequent complication and an important cause of death in patients with paralysis. We aimed to investigate predictors of pulmonary embolism (PE) and the impact of PE on the survival of patients with paralysis in comparison to those with deep venous thrombosis or thrombophlebitis (DVT). Methods: Patients were selected by screening the German nationwide inpatient sample (2005–2017) for paralysis, and were stratified for venous thromboembolism (VTE) and the VTE-sub-entity PE (ICD-code I26). Impact of PE on mortality and predictors for PE were analyzed. Results: Overall, 7,873,769 hospitalizations of patients with paralysis were recorded in Germany 2005–2017, of whom 1.6% had VTE and 7.0% died. While annual hospitalizations increased (2005: 520,357 to 2017: 663,998) (β 12,421 (95% CI 10,807 to 14,034), *p* < 0.001), in-hospital mortality decreased from 7.5% to 6.7% (β −0.08% (95% CI −0.10% to −0.06%), *p* < 0.001). When focusing on 82,558 patients with paralysis hospitalized due to VTE (51.8% females; 58.3% aged ≥ 70 years) in 2005–2017, in-hospital mortality was significantly higher in patients with paralysis and PE than in those with DVT only (23.8% vs. 6.3%, *p* < 0.001). Cancer (OR 2.18 (95% CI 2.09–2.27), *p* < 0.001), heart failure (OR 1.83 (95% CI 1.76–1.91), *p* < 0.001), COPD (OR 1.63 (95% CI 1.53–1.72), *p* < 0.001) and obesity (OR 1.42 (95% CI 1.35–1.50), *p* < 0.001) were associated with PE. PE (OR 4.28 (95% CI 4.07–4.50), *p* < 0.001) was a strong predictor of in-hospital mortality. Conclusions: In Germany, annual hospitalizations of patients with paralysis increased in 2005–2017, in whom VTE and especially PE substantially affected in-hospital mortality. Cancer, heart failure, COPD, obesity and acute paraplegia were risk factors of PE.

## 1. Introduction

Paralysis is categorized as a moderate to strong risk factor for the development of venous thromboembolism (VTE) [1,2,3,4,5,6,7,8,9].It has been estimated that the incidence of deep venous thrombosis in paralysis and stroke patients exceeds even the incidence of deep venous thrombosis in patients with general surgeries, and might be similar to the high rates occurring after hip and knee joint arthroplasty [1,3,5,6,7,10,11].

Without VTE prophylaxis, several studies revealed that up to 75% of the stroke patients with hemiplegia or paralysis develop deep venous thrombosis. [1,5,6] Overall, two-thirds of these deep venous thrombosis events are asymptomatic and occur below the knee level [1]. Importantly, the risk for deep venous thrombosis correlated with the degree of paralysis, with predilection for the paralyzed leg [1,6].

In about 65% of all cases, pulmonary embolism (PE) is the consequence of deep venous thrombosis, rather than a separate clinical disease entity [12,13,14]. For PE without underlying deep venous thrombosis (isolated PE), data from different studies suggest an important role of co-morbidities, including cancer [15], atrial fibrillation [16,17], myocardial infarction [16] and heart failure [16,17] in the pathogenesis of central thrombus formation. Although major trauma, surgery, immobilization, cancer and thrombophilia are well-recognized traditional risk factors for VTE [9,18], several chronic inflammatory diseases, such as inflammatory bowel disease, psoriasis, rheumatoid arthritis, systemic lupus erythematosus, Sjogren syndrome, celiac disease, systemic sclerosis and dermatomyositis, as well as polymyositis, were associated with an increased risk of VTE [18,19,20,21,22,23,24,25,26,27].

Since the incidence of VTE, comprising deep venous thrombosis and PE, is high in patients with paralysis [3,5] and the VTE sub-entity PE, especially, is accompanied by a high risk of an adverse in-hospital course [4,9,11,20] it is of outstanding interest to identify factors that result in or are associated with the development of the life-threatening complication of an acute PE.

## 2. Methods and Patients

For our present study, we analyzed the nationwide inpatient sample of Germany with diagnosis-related group (DRG) statistics. As implemented in the year 2004, patients’ diagnoses must be coded in Germany according to the ICD-10-GM (International Classification of Diseases, 10th Revision with German Modification) and patients’ diagnostical as well as surgical and interventional approaches must be coded according to OPS codes (Operationen- und Prozedurenschlüssel), and the hospitals send these codes to the Institute for the Hospital Remuneration System to obtain their remuneration. In parallel, the Federal Statistical Office of Germany (Statistisches Bundesamt) collects and assesses these data for the German nationwide inpatient sample, which was used for the present analysis. [7,28,29].

The Research Data Center (RDC) of the Federal Statistical Office and the Statistical Offices of the federal states in Wiesbaden (Germany) ran the study analyses on our behalf. For the trend analyses of hospitalizations of patients with paralysis (with and without VTE) 2005–2017, we requested from the RDC an analysis of total annual numbers of hospitalizations of patients with paralysis (ICD codes G80-G83), with and without VTE (ICD codes I26, I80-I82), and among these, deceased patients. Out of these data, rate of VTE and in-hospital mortality of hospitalized patients with paralysis were calculated (Statistisches Bundesamt, DEStatis, source: DRG-Statistik, Sonderauswertung des Statistischen Bundesamtes) [30].

**Figure 1:**
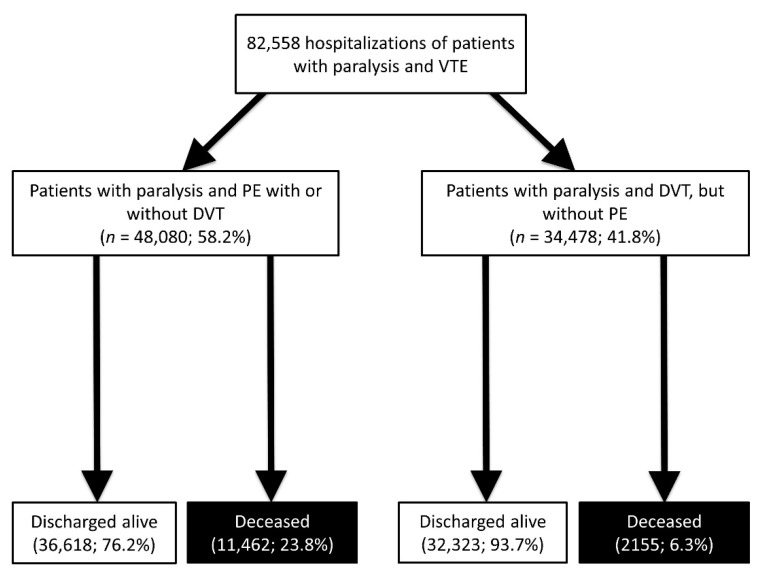
Flow diagram.

For the second part of the statistical analysis, and therefore our further analysis in detail, we focused on an excerpt of these patients with paralysis in Germany and selected all hospitalized patients with paralysis (ICD codes G80-G83) in co-prevalence with VTE (main diagnosis of deep vein thrombosis or thrombophlebitis (DVT, ICD-codes I80, I81, I82) and/or main or secondary diagnosis of PE (ICD-code I26)) in Germany between 2005 and 2017; these patients were stratified for presence of PE (ICD-code I26) (source: RDC of the Federal Statistical Office and the Statistical Offices of the federal states, DRG Statistics 2005–2017, own calculations) (Figure 1). The main diagnosis of a patient was defined as the diagnosis mainly responsible for hospitalization documented by the discharging physician [31]. All analyzed VTE risk factors, comorbidities and adverse in-hospital events were defined and coded according to the ICD-10-GM.

### 2.1. Study Endpoints

The primary study endpoints comprised (I) the presence of PE and (II) death from all causes during in-hospital stay (in-hospital death).

### 2.2. Ethical Aspects and Study Oversight

Since direct access by the investigators to the data of individual patients was not given in this present study, and only the access to summarized results provided by the RDC was the basis of these study results, approval by an ethics committee as well as an informed consent were not required, in accordance with German law [32].

## 3. Statistics

Descriptive statistics for the relevant comparisons of (i) patients with paralysis in co-prevalence with isolated DVT (without PE) and patients with paralysis in co-prevalence with PE (with or without DVT) and (ii) patients with paralysis in co-prevalence with VTE stratified for decease during hospitalization (in-hospital death) were shown as median and interquartile range (IQR), or presented as absolute numbers and corresponding percentages. In case of continuous variables, these variables/parameters were compared with the help of the Wilcoxon–Whitney U test, and categorical variables were tested with Fisher’s exact or chi [2] test, as appropriate [32,33].

Logistic regression models were calculated to investigate (i) associations between patients’ characteristics and adverse events, and presence of PE in patients with paralysis in co-prevalence with any VTE, in order to identify parameters that are accompanied by an association to PE in patients with paralysis and VTE, and (ii) associations between patients’ characteristics and adverse events, as well as presence of PE and in-hospital death in patients with paralysis in co-prevalence with VTE. These results of the logistic regressions were presented as Odds Ratios (OR) and 95% confidence intervals (CI). To ensure that the results of the logistic regression analyses were not substantially influenced by biasing factors, and therefore guarantee a broad independence from different cofactors, we adjusted the multivariate logistic regression models with the following parameters: age, gender, cancer, coronary artery disease, heart failure, chronic obstructive pulmonary disease (COPD), arterial hypertension, acute and chronic kidney disease, diabetes mellitus, atrial fibrillation/flutter, smoking and hyperlipidaemia.

We worked and operated with the software SPSS^®^ (IBM Corp. Released 2011. IBM SPSS Statistics for Windows, Version 20.0. IBM Corp: Armonk, NY, USA) for the computerized analysis. Only *p* values of <0.05 (two-sided) were considered to be statistically significant.

## 4. Results

### 4.1. Time-Trends Regarding Hospitalizations of Patients with Paralysis with and without VTE

**Figure 2:**
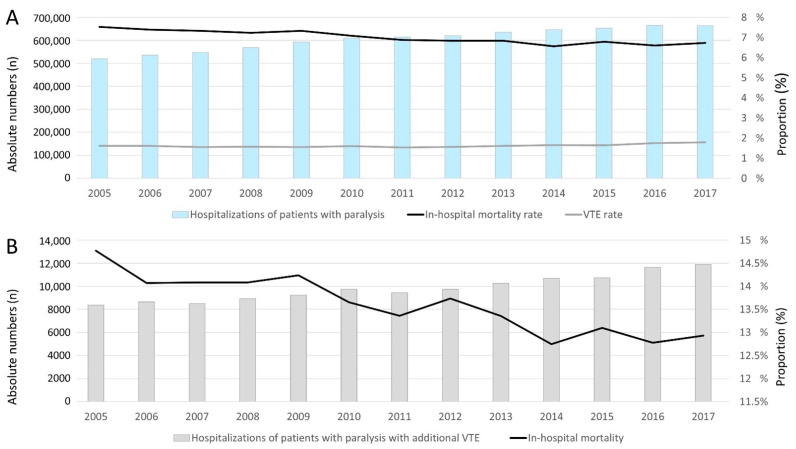
Temporal trends regarding hospitalizations of patients with paralysis in 2015–2017 in Germany (**A**): All hospitalizations of patients with paralysis (blue bars), VTE rate (grey line) and in-hospital death rate (black line). (**B**): Annual numbers of patients with paralysis and VTE (grey bars) and their in-hospital mortality rate (black line).

Overall, 7,873,769 hospitalizations of patients with paralysis were recorded in the German nationwide inpatient sample between 2005 and 2017. In total, 550,058 (7.0%) patients died during the observational period. While the annual hospitalizations increased from 520,357 in the year 2005 to 663,998 in the year 2017 (β 12,421.12 per year [95% CI 10,807.73 to 14,034.51], *p* < 0.001), the in-hospital mortality decreased from 7.5% in 2005 to 6.7% in 2017 (β −0.08% per year (95% CI −0.10% to −0.06%), *p* < 0.001) (Figure 2A). In total, 127,766 hospitalizations of patients with paralysis were counted with VTE (1.6% of all hospitalizations of all patients with paralysis). In parallel with the annual number of hospitalizations of patients with paralysis, the hospitalizations of patients with paralysis and VTE increased from 8360 in 2005 to 11,890 in 2017 (β 287.87 per year (95% CI 242.39 to 333.35), *p* < 0.001). In contrast, the proportion of patients with paralysis and VTE was widely constant with a small uptrend (1.6% in 2005 and 1.8% in 2017) (β 0.014% (95% CI 0.004% to 0.023%), *p* = 0.008) (Figure 2B). The in-hospital death rate was 13.5% in patients with paralysis and VTE, and considerably higher than in patients with paralysis only (in-hospital death rate: 7.0%). Encouragingly, the in-hospital death rate of patients with paralysis and VTE decreased from 14.8% in the year 2005 to 12.9% in 2017 (β −0.15% (95% CI −0.19% to −0.11%), *p* < 0.001) (Figure 2B).

### 4.2. Comparison of Patients with Paralysis in Co-Prevalence with Isolated DVT (without PE) and Patients with Paralysis in Co-Prevalence with PE (with or without DVT)

The German nationwide inpatient sample comprised 82,558 patients with paralysis hospitalized due to DVT or with PE (51.8% females; 58.3% aged ≥ 70 years) between 2005 and 2017. Among these patients with paralysis, 48,080 (58.2%) were diagnosed with PE and 34,478 patients (41.8%) with isolated DVT (Figure 1). In total, 13,617 (16.5%) of the patients with paralysis and VTE died during hospitalization (Figure 1).

Patients with paralysis and PE were slightly younger than those with paralysis and isolated DVT. The classical VTE risk factors, cancer and surgery, were substantially more common in patients with PE (Table 1). In addition, comorbidities, such as heart failure and COPD, were more prevalent in patients with paralysis and PE in comparison to those with paralysis and isolated DVT. In contrast, peripheral artery disease, arterial hypertension and hyperlipidaemia were more frequent in patients with paralysis and DVT only, and the groups did not differ regarding inflammatory bowel disease.

While the paralysis was related to an acute stroke event in 59.8% (20,617) of the patients with co-prevalence of paralysis with isolated DVT, stroke was substantially less prevalent in patients with paralysis and PE, at 34.2% (16,456) (*p* < 0.001). While the acute paraplegia sub-entities were more prevalent in patients with paralysis and PE than in those with DVT only, infantile cerebral palsy was more frequent in patients with paralysis and DVT only, compared to those with PE (Table 1).

As expected, the risk stratification markers of right ventricular dysfunction (RVD), myocardial injury, syncope and shock occurred more often in patients with PE compared to those with DVT but without PE (Table 1).

All-cause in-hospital mortality was significantly higher in patients with paralysis and PE than in those with DVT only (23.8% vs. 6.3%, *p* < 0.001). In line, bleeding events, such as intracerebral bleeding, gastro-intestinal bleeding and the necessity for transfusion of blood constituents were higher in patients with paralysis and PE (Table 1).

### 4.3. Risk Factors for Occurrence of PE in Patients with Paralysis and VTE

All investigated acute forms of paraplegia, such as acute complete or incomplete paraplegia (OR 9.80 (95% CI 7.89–12.18), *p* < 0.001), acute complete paraplegia (OR 7.55 (95% CI 5.15–11.06), *p* < 0.001) and acute incomplete paraplegia were strongly associated with PE (OR 10.77 (95% CI 8.29–14.01), *p* < 0.001) (Table 2). Cancer (OR 2.18 (95% CI 2.09–2.27), *p* < 0.001), heart failure (OR 1.83 (95% CI 1.76–1.91), *p* < 0.001), COPD (OR 1.63 (95% CI 1.53–1.72), *p* < 0.001) and obesity (OR 1.42 (95% CI 1.35–1.50), *p* < 0.001) were important and independent risk factors for a PE in patients with paralysis (and VTE) (Table 2). In contrast, peripheral artery disease, arterial hypertension and hyperlipidaemia, as well as stroke, were independently associated with paralysis in co-prevalence with DVT but without PE (Table 2).

### 4.4. Differences between Patients with Paralysis and VTE Who Died during Hospitalization and Those Who Were Discharged Alive

Overall, 13,617 (16.5%) patients with paralysis and VTE died during their in-hospital stay. The deceased patients with paralysis and VTE were in median 2 years older and more often had cancer, heart failure, coronary artery disease, COPD, acute and chronic kidney disease and diabetes mellitus than those who were discharged alive (Table 3). PE was substantially more common in deceased patients (84.2% vs. 53.1%, *p* < 0.001). Bleeding events, such as intracerebral bleeding, gastro-intestinal bleeding and necessity of transfusion of blood constituents, as well as shock, were more frequent in patients who died during the in-hospital course (Table 3).

### 4.5. Predictors of In-Hospital Death in Patients with Paralysis and VTE

PE (OR 4.28 (95% CI 4.07–4.50), *p* < 0.001) and RVD (OR 4.84 (95% CI 4.64–5.05), *p* < 0.001) as well as shock (OR 5.06 (95% CI 4.69–5.46), *p* < 0.001 were the strongest independent PE-specific predictors of in-hospital mortality in patients with paralysis and VTE (Table 4). Age ≥70 years (OR 1.35 (95% CI 1.29–1.41), *p* < 0.001), cancer (OR 2.18 (95% CI 2.08–2.28), *p* < 0.001), heart failure (OR 1.50 (95% CI 1.43–1.57), *p* < 0.001), acute and chronic kidney disease (OR 1.62 (95% CI 1.55–1.69), *p* < 0.001), stroke (OR 1.32 (95% CI 1.27–1.37), *p* < 0.001) and all bleeding events were independently associated with increased mortality (Table 4).

## 5. Discussion

The main results of our present study can be summarized as follows:

1. In total, 7,873,769 hospitalizations of patients with paralysis were recorded in the German nationwide inpatient sample with an increase in the total annual number from 2005 to 2017.
2. Among these patients, 7.0% died during the observational period, and this rate decreased during the observational period.
3. Overall, 1.6% of all hospitalized patients with paralysis had or developed an event of VTE.
4. The in-hospital mortality rate was 13.5% in patients with paralysis and VTE.
5. Cancer, heart failure, COPD, and obesity were important and independent risk factors for PE in patients with paralysis and VTE, whereas peripheral artery disease, arterial hypertension and hyperlipidaemia as well as stroke were independently associated with paralysis in co-prevalence with DVT only.
6. Acute forms of paraplegia were strongly associated with PE in paralysis.
7. Independent predictors of in-hospital death in patients with paralysis and VTE were PE, shock, age ≥ 70 years, cancer, heart failure, acute and chronic kidney disease, stroke as well as all bleeding events.

### 5.1. Prevalence of Hospitalizations of Patients with Paralysis

The prevalence of paralysis was estimated as 1.7% in the US population in the year 2013, which represents more than 5 million people in the US [34]. In accordance with those data, the German inpatient sample revealed a high number of hospitalizations of patients with paralysis (7,873,769 hospitalizations) in the observational period 2005–2017 as well. In Germany, the annual hospitalizations of patients with paralysis increased from 2005 to 2017, which is in accordance with an increasing number of disabling strokes based on the data of the Global Burden of Disease 2013 Study [35].

### 5.2. Main Causes of Paralysis

Stroke was the leading cause of paralysis in literature, followed by spinal cord injuries [34]. Remarkably, almost 72% of persons with paralysis were younger than 65 years [34]. Similarly, our study showed a high stroke rate (44.9%) in patients with paralysis and VTE. Notably, stroke rate was lower in patients with paralysis and PE in comparison to those with paralysis and isolated DVT.

### 5.3. Prevalence of VTE in Hospitalizations of Patients with Paralysis

Paralysis is a moderate to strong risk factor for the development of VTE [8,9]. In patients with paralysis it is of particular interest that due to disabilities and immobilization, thromboembolic prophylaxis seems unfortunately to be less effective than in surgical patients [3].

Overall, 1.6% of all hospitalized patients with paralysis developed an event of VTE. As known from other studies, the prevalence of DVT (symptomatic and asymptomatic) differs in surgical and non-surgical medicine without VTE prophylaxis—between 10% and 20% in internal medicine diseases, distinctly higher in stroke (20–50%) and polytrauma (40–80%), and highest in patients with spinal cord injury (60–80%) [36]. Other studies reported that without VTE prophylaxis, approximately 50–75% of stroke patients with hemiplegia or paralysis develop DVT [1,5,6]. Thus, VTE is a common complication in spinal cord injury and stroke with resulting paresis [3,5,7,8,9,10,11,36]. Among traumatic injuries, traumatic spine injuries of the spinal cord and/or the cauda equina are connected with the highest VTE risk [36]. Remarkably, the primary reasons for this high risk are related to the failure of the muscle pump driven by the paresis in combination with a presumed transient hypercoagulative phase, and, particularly, endothelial damage [36]. Moreover, decoupling from supraspinal control seems to be another important contributing prothrombotic factor [36].

Besides leg oedema caused by post-thrombotic syndrome and hemorrhage as a complication of the required anticoagulation, VTE in particular is related to poor outcome in patients with paralysis [36]. DVT is one of the major complications in patients with paralysis because of Virchow’s triad, which comprises stasis, endothelial cell injury and hypercoagulability [37]. PE occurs in the large majority of cases when a thrombotic material from DVT breaks loose and travels through the bloodstream to the pulmonary artery bed [7,9,14], and is the most important and life-threatening manifestation of VTE [36]. Since DVT is the most common VTE entity in patients with paralysis, it is of outstanding interest that the majority of these DVT events is asymptomatic [1,12,13,14]. Thus, PE might be the first recognized manifestation of VTE, although the underlying previously developed DVT event might be undetected or overlooked, which can impede a life-saving early start to anticoagulant treatment [1,12,13,14]

### 5.4. PE Is Related to a High Risk of In-Hospital Mortality in Patients with Paralysis

Notably, in patients with stroke or paralysis, PE occurs in approximately one-fifth of these patients [5], and PE events are affected by aggravated outcomes with high morbidity and mortality [5,7,9,32]. Therefore, since the incidence of DVT is high in patients with paralysis [3,5] the risk for PE increases with DVT rate and PE events are accompanied by a high risk of death in the short-term [4,9,11,20], it is of major interest to identify factors associated with development of the life-threatening complication of acute PE.

### 5.5. Predictors of PE in Patients with Paralysis

Our study identified cancer, heart failure, COPD, obesity and acute and chronic kidney diseases as important and independent risk factors for the occurrence of PE in patients with paralysis and VTE. These findings are in broad agreement with previous study results reporting the important roles of cancer [15], atrial fibrillation [16,17] myocardial infarction [16] and heart failure [16,17] in the pathogenesis of PE. In contrast, peripheral artery disease, arterial hypertension and hyperlipidaemia were independently associated with DVT without PE in patients with paralysis and VTE. In this context, a link between atherosclerosis and DVT was demonstrated in several studies [33,38,39,40,41,42]. Prandoni et al. reported a higher frequency of carotid plaques as an indicator of atherosclerosis in patients with previous idiopathic DVT in comparison to those without DVT [39]. Other studies confirmed the association between atherosclerosis and atherosclerotic risk factors, such as hyperlipidaemia and VTE [33,41,42,43,44,45]. Acute forms of paraplegia were strongly associated with PE in paralysis, which is in accordance with published literature [46].

### 5.6. In-Hospital Mortality Rate of Patients with Paralysis

The reported in-hospital mortality rate of patients hospitalized with paralysis was 7.0%, and thus in line to other studies reporting in-hospital mortality rates due to acute stroke as between 5% and 14% [7,47,48,49], as well as death due to spinal cord injury ranging between 2% and 24% [50,51]. In this context, it is important to mention that the results of hospitalized patients with paralysis in our study do not focus only on these acute forms of paralysis, but also include patients with long-lasting non-acute forms of paralysis, which might explain the lower in-hospital mortality in our study.

### 5.7. Impact of VTE on Survival of Patients with Paralysis

As expected, VTE is a frequent and harmful complication in patients with paralysis [1,2,3,4,5,6,7], and the mortality rate in hospitalizations of patients with paralysis was substantially higher in presence of VTE. In addition, at 13.5%, the in-hospital mortality rate in patients with paralysis and VTE was 1.9-fold higher than that of patients with paralysis (7.0%) but without VTE.

### 5.8. Independent Predictors of In-Hospital Death in Patients with Paralysis and VTE

In accordance with the literature, we identified that higher age, cancer, heart failure, acute and chronic kidney disease, stroke, all bleeding events and PE are independent predictors of in-hospital death in patients with paralysis and VTE [7,9,32,52,53,54,55,56]. Notably, PE was associated with a 4.3-fold risk of death during hospitalization, which is in line with previous studies confirming the marked impact of PE and respiratory complications on in-hospital mortality [7,57,58,59].

In this context, it must also be considered that the number of PE-related deaths may even be underestimated in paralytic patients, since approximately half of the PE events in these patients occur as sudden death, and therefore might not be recognized as PE [3,4,7].

## 6. Limitations

There are certain limitations of our present study which merit consideration: Firstly, as aforementioned, study results were based on ICD and OPS codes, which might be subject to underreporting or miscoding. Secondly, detailed baseline data, including patients’ concomitant medications, cardiac troponin plasma concentrations, and echocardiographic parameters, and outcome data, such as major bleeding, were not available in the German nationwide inpatient sample. Third, exact timing of acute adverse events during hospitalizations could not be determined. Fourthly, information on the exact cause of death cannot be obtained from the German nationwide inpatient sample [32].

## 7. Conclusions

In Germany, the annual hospitalizations of patients with paralysis increased from 2005 to 2017. Among these inpatients, 7.0% died and 1.6% developed an event of VTE. VTE affected the in-hospital mortality rate of patients with paralysis substantially. Cancer, heart failure, COPD, obesity and acute paraplegia were important and independent risk factors for acute PE. PE was associated with 4.3-fold increase in-hospital mortality.

## Figures and Tables

**Table 1:** Patients’ characteristics, medical history, presentation, treatments and adverse in-hospital events of 82,558 patients with paralysis (ICD codes G80-G83) in co-prevalence with isolated DVT (without PE) (ICD-codes I26, I80, I81, I82) in comparison to patients with paralysis and PE (with or without DVT) (ICD-code I26).

Parameters	Patients with Paralysis and PE with or without DVT (*n* = 48,080; 58.2%)	Patients with Paralysis and Isolated DVT (without PE) (*n* = 34,478; 41.8%)	*p*-Value
Median age (years)	73.00 (62.00–80.00)	74.00 (63.00–81.00)	<0.001
Age ≥ 70 years	27,337 (56.9%)	20,781 (60.3%)	<0.001
Gender (females)	24,520 (51.0%)	18,244 (52.9%)	<0.001
In-hospital stay (days)	15 (8–28)	12 (7–22)	<0.001
Obesity	4586 (9.5%)	2465 (7.1%)	<0.001
Acute paraplegia and infantile cerebral palsy			
Acute complete or incomplete paraplegia	1337 (2.8%)	88 (0.3%)	<0.001
Acute complete paraplegia	354 (0.7%)	29 (0.1%)	<0.001
Acute incomplete paraplegia	1002 (2.1%)	60 (0.2%)	<0.001
Infantile cerebral palsy	346 (0.7%)	312 (0.9%)	0.003
VTE risk factors			
Surgery during in-hospital stay	30,237 (62.9%)	18,188 (52.8%)	<0.001
Cancer	9589 (19.9%)	3509 (10.2%)	<0.001
Inflammatory bowel disease	273 (0.6%)	189 (0.5%)	0.710
Thrombophilia	411 (0.8%)	271 (0.8%)	0.282
Comorbidities			
Heart failure	11,512 (23.9%)	5162 (14.9%)	<0.001
Coronary artery disease	7479 (15.6%)	5198 (15.1%)	0.060
Cardiomyopathy	711 (1.5%)	385 (1.1%)	<0.001
Peripheral artery disease	1994 (4.1%)	1814 (5.3%)	<0.001
Chronic obstructive pulmonary disease	4334 (9.0%)	1923 (5.6%)	<0.001
Arterial hypertension	24,310 (50.6%)	19,732 (57.2%)	<0.001
Hyperlipidaemia	7731 (16.1%)	8174 (23.7%)	<0.001
Acute and chronic kidney disease	11,091 (23.1%)	6459 (18.7%)	<0.001
Diabetes mellitus	12,468 (25.9%)	9141 (26.5%)	0.061
Deep venous thrombosis or thrombophlebitis	14,224 (29.6%)	34,478 (100.0%)	<0.001
Atrial fibrillation/flutter	11,775 (24.5%)	8568 (24.9%)	0.236
Risk stratification markers			
Right ventricular dysfunction	14,702 (30.6%)	0 (0.0%)	<0.001
Syncope	876 (1.8%)	255 (0.7%)	<0.001
Adverse events during the in-hospital stay			
All cause in-hospital death	11,462 (23.8%)	2155 (6.3%)	<0.001
Stroke	16,456 (34.2%)	20,617 (59.8%)	<0.001
Myocardial injury	1742 (3.6%)	768 (2.2%)	<0.001
Shock	2611 (5.4%)	520 (1.5%)	<0.001
Cardio-pulmonary resuscitation	4087 (8.5%)	329 (1.0%)	<0.001
Intracerebral bleeding	3442 (7.1%)	1094 (3.2%)	<0.001
Subarachnoid hemorrhage	592 (1.2%)	462 (1.3%)	0.169
Gastro-intestinal bleeding	1026 (2.1%)	469 (1.4%)	<0.001
Reperfusion treatments and transfusion of blood constituents			
Systemic thrombolysis	3086 (6.4%)	2174 (6.3%)	0.512
Transfusion of blood constituents	8679 (18.1%)	3202 (9.3%)	<0.001

Abbreviations: VTE indicates venous thromboembolism, PE = pulmonary embolism, DVT = deep venous thrombosis or thrombophlebitis.

**Table 2:** Factors associated with pulmonary embolism in patients with paralysis in coprevalence with venous thromboembolism.

	Uni-Variate Regression Model		Multi-Variate Regression Model	
Parameters	OR (95% CI)	*p*-Value	OR (95% CI)	*p*-Value
Age	0.995 (0.994–0.996)	<0.001	0.994 (0.993–0.996)	<0.001
Age ≥ 70 years	0.87 (0.85–0.89)	<0.001	0.87 (0.84–0.90)	<0.001
Gender (females)	0.93 (0.90–0.95)	<0.001	0.96 (0.94–0.99)	0.011
Obesity	1.37 (1.30–1.44)	<0.001	1.42 (1.35–1.50)	<0.001
Acute paraplegia and infantile cerebral Palsy				
Acute complete or incomplete paraplegia	11.18 (9.00–13.87)	<0.001	9.80 (7.89–12.18)	<0.001
Acute complete paraplegia	8.81 (6.03–12.87)	<0.001	7.55 (5.15–11.06)	<0.001
Acute incomplete paraplegia	12.21 (9.40–15.84)	<0.001	10.77 (8.29–14.01)	<0.001
Infantile cerebral palsy	0.79 (0.68–0.93)	0.003	0.73 (0.62–0.85)	<0.001
VTE risk factors				
Surgery during in-hospital stay	1.52 (1.48–1.56)	<0.001	1.44 (1.40–1.48)	<0.001
Cancer	2.20 (2.11–2.29)	<0.001	2.18 (2.09–2.27)	<0.001
Inflammatory bowel disease	1.04 (0.86–1.25)	0.710	0.99 (0.82–1.20)	0.918
Thrombophilia	1.09 (0.93–1.27)	0.282	1.04 (0.89–1.22)	0.642
Comorbidities				
Heart failure	1.79 (1.72–1.85)	<0.001	1.83 (1.76–1.91)	<0.001
Coronary artery disease	1.04 (1.00–1.08)	0.060	1.01 (0.97–1.05)	0.617
Cardiomyopathy	1.33 (1.17–1.51)	<0.001	1.06 (0.93–1.20)	0.414
Chronic obstructive pulmonary disease	1.68 (1.59–1.77)	<0.001	1.63 (1.53–1.72)	<0.001
Hyperlipidaemia	0.62 (0.60–0.64)	<0.001	0.65 (0.63–0.68)	<0.001
Arterial hypertension	0.76 (0.74–0.79)	<0.001	0.88 (0.86–0.91)	<0.001
Acute and chronic kidney disease	1.30 (1.26–1.35)	<0.001	1.24 (1.20–1.29)	<0.001
Diabetes mellitus	0.97 (0.94–1.00)	0.061	0.97 (0.94–1.01)	0.123
Peripheral artery disease	0.78 (0.73–0.83)	<0.001	0.75 (0.70–0.80)	<0.001
Atrial fibrillation/flutter	0.98 (0.95–1.01)	0.236	0.96 (0.93–1.00)	0.034
Stroke (ischemic and hemorrhagic)	0.35 (0.34–0.36)	<0.001	0.36 (0.35–0.37)	<0.001

**Table 3:** Characteristics, medical history, presentation, treatments and adverse in-hospital events of 82,558 patients with paralysis (ICD codes G80-G83) in co-prevalence with venous thromboembolism (ICD-codes I26, I80, I81, I82) and stratified for in-hospital death.

Parameters	Patients with Paralysis and VTE, Who Died IN-Hospital (*n* = 13,617; 16.5%)	Patients with Paralysis and VTE, Who Were Discharged Alive (*n* = 68,941; 83.5%)	*p*-Value
Median age (years)	75.0 (66.0–82.0)	73.0 (62.0–80.0)	<0.001
Age ≥ 70 years	8801 (64.6%)	39,317 (57.0%)	<0.001
Gender (females)	6953 (51.1%)	35,811 (51.9%)	0.059
In-hospital stay (days)	10 (4–21)	15 (8–27)	<0.001
Obesity	997 (7.3%)	6054 (8.8%)	<0.001
Paraplegia and infantile cerebral palsy			
Acute complete or incomplete paraplegia	255 (1.9%)	1170 (1.7%)	0.141
Acute complete paraplegia	85 (0.6%)	298 (0.4%)	0.002
Acute incomplete paraplegia	173 (1.3%)	889 (1.3%)	0.880
Infantile cerebral palsy	95 (0.7%)	563 (0.8%)	0.160
VTE risk factors			
Surgery during in-hospital stay	7635 (56.1%)	40,790 (59.2%)	<0.001
Cancer	3340 (24.5%)	9758 (14.2%)	<0.001
Inflammatory bowel disease	79 (0.6%)	383 (0.6%)	0.710
Thrombophilia	78 (0.6%)	604 (0.9%)	<0.001
Comorbidities			
Heart failure	3926 (28.7%)	12,748 (18.4%)	<0.001
Coronary artery disease	2358 (17.3%)	10,319 (15.0%)	<0.001
Cardiomyopathy	230 (1.7%)	866 (1.2%)	<0.001
Chronic obstructive pulmonary disease	1298 (9.5%)	4959 (7.2%)	<0.001
Arterial hypertension	6420 (47.1%)	37,622 (54.6%)	<0.001
Hyperlipidaemia	1732 (12.7%)	14,173 (20.6%)	<0.001
Acute and chronic kidney disease	4205 (30.9%)	13,345 (19.4%)	<0.001
Diabetes mellitus	3995 (29.3%)	17,614 (25.5%)	<0.001
Pulmonary embolism	11,462 (84.2%)	36,618 (53.1%)	<0.001
Deep venous thrombosis or thrombophlebitis	4029 (29.6%)	44,673 (64.8%)	<0.001
Stroke	6560 (48.2%)	30,513 (44.3%)	<0.001
Myocardial injury	727 (5.3%)	1783 (2.6%)	<0.001
Atrial fibrillation/flutter	4026 (29.6%)	16,317 (23.7%)	<0.001
Intracerebral bleeding	1034 (7.6%)	3502 (5.0%)	<0.001
Subarachnoid hemorrhage	170 (1.2%)	884 (1.3%)	0.770
Gastro-intestinal bleeding	411 (3.0%)	1084 (1.6%)	<0.001
Transfusion of blood constituents	3233 (23.7%)	8648 (12.5%)	<0.001
Shock	1557 (11.4%)	1574 (2.3%)	<0.001

Abbreviations: VTE indicates venous thromboembolism, PE = pulmonary embolism, DVT = deep venous thrombosis or thrombophlebitis.

**Table 4:** Risk factors for in-hospital death in patients with paralysis in co-prevalence with venous thromboembolism.

	Uni-Variate Regression Model		Multi-Variate Regression Model	
Parameters	OR (95% CI)	*p*-Value	OR (95% CI)	*p*-Value
Age	1.016 (1.015–1.017)	<0.001	1.016 (1.014–1.017)	<0.001
Age ≥ 70 years	1.38 (1.33–1.43)	<0.001	1.35 (1.29–1.41)	<0.001
Gender (females)	0.97 (0.93–1.00)	0.059	0.90 (0.87–0.94)	<0.001
Obesity	0.82 (0.77–0.88)	<0.001	0.89 (0.82–0.95)	0.001
Paraplegia and infantile cerebral palsy				
Acute complete or incomplete paraplegia	1.11 (0.97–1.27)	0.141	0.99 (0.86–1.14)	0.987
Acute complete paraplegia	1.45 (1.14–1.85)	0.003	1.31 (1.02–1.68)	0.037
Acute incomplete paraplegia	0.99 (0.84–1.16)	0.880	0.88 (0.74–1.04)	0.129
Infantile cerebral palsy	0.86 (0.69–1.06)	0.160	1.25 (1.00–1.57)	0.050
VTE risk factors				
Surgery during in-hospital stay	0.88 (0.85–0.91)	<0.001	0.83 (0.80–0.86)	<0.001
Cancer	1.97 (1.89–2.06)	<0.001	2.18 (2.08–2.28)	<0.001
Inflammatory bowel disease	1.05 (0.82–1.34)	0.710	1.18 (0.92–1.51)	0.199
Thrombophilia	0.65 (0.52–0.83)	<0.001	0.78 (0.61–0.99)	0.043
Comorbidities				
Heart failure	1.79 (1.72–1.87)	<0.001	1.50 (1.43–1.57)	<0.001
Coronary artery disease	1.19 (1.13–1.25)	<0.001	1.04 (0.98–1.09)	0.185
Cardiomyopathy	1.35 (1.17–1.57)	<0.001	1.17 (1.00–1.36)	0.048
Chronic obstructive pulmonary disease	1.36 (1.28–1.45)	<0.001	1.21 (1.13–1.29)	<0.001
Hyperlipidaemia	0.56 (0.53–0.59)	<0.001	0.58 (0.54–0.61)	<0.001
Arterial hypertension	0.74 (0.72–0.77)	<0.001	0.76 (0.73–0.79)	<0.001
Acute and chronic kidney disease	1.86 (1.79–1.94)	<0.001	1.62 (1.55–1.69)	<0.001
Diabetes mellitus	1.21 (1.16–1.26)	<0.001	1.13 (1.09–1.18)	<0.001
Pulmonary embolism	4.70 (4.47–4.93)	<0.001	4.28 (4.07–4.50)	<0.001
Peripheral artery disease	1.46 (1.35–1.58)	<0.001	1.31 (1.21–1.43)	<0.001
Atrial fibrillation/flutter	1.35 (1.30–1.41)	<0.001	1.17 (1.12–1.22)	<0.001
Stroke (ischemic and hemorrhagic)	1.17 (1.13–1.22)	<0.001	1.32 (1.27–1.37)	<0.001
Risk stratification parameters				
Syncope	1.08 (0.92–1.26)	0.340	0.98 (0.84–1.15)	0.790
Right ventricular dysfunction in PE	4.94 (4.75–5.15)	<0.001	4.84 (4.64–5.05)	<0.001
Shock	5.46 (5.08–5.87)	<0.001	5.06 (4.69–5.46)	<0.001
Adverse in-hospital events				
Intracerebral bleeding	1.54 (1.43–1.65)	<0.001	1.85 (1.72–1.99)	<0.001
Subarachnoid bleeding	0.98 (0.83–1.15)	0.770	1.39 (1.17–1.64)	<0.001
Gastro-intestinal bleeding	1.95 (1.74–2.19)	<0.001	1.66 (1.47–1.87)	<0.001
Transfusion of blood constituents	2.16 (2.07–2.26)	<0.001	1.90 (1.81–1.99)	<0.001

## Data Availability

The data were provided from the Federal Statistical Office of Germany (Statistisches Bundesamt, DEStatis) (source: RDC of the Federal Statistical Office and the Statistical Offices of the federal states, DRG Statistics 2005–2017, and own calculations).

## Abbreviations

CI	Confidence Interval
DVT	Deep vein thrombosis and thrombophlebitis of the leg veins
DRG	Diagnosis-Related Groups
ICD	International Classification of Disease
IQR	Interquartile range
OPS	Surgery and interventional procedure keys (Operationen- und Prozedurenschlüssel)
OR	Odds ratio
PE	Pulmonary Embolism
RVD	Right ventricular dysfunction
VTE	Venous thromboembolism
